# Supplementary material for: Diterpene Resin Acids and Olefins in Calabrian Pine (Pinus nigra subsp. laricio (Poiret) Maire) Oleoresin: GC-MS Profiling of Major Diterpenoids in Different Plant Organs, Molecular Identification and Expression Analysis of Diterpene Synthase Genes
Source: Plants (Basel). 2021 Nov 5;10(11):2391. doi: 10.3390/plants10112391 (PMC8622628; doi:10.3390/plants10112391)
Supplement: Supplementary file 1 [file plants-10-02391-s001.zip › plants-1418073-supplementary.pdf]

Additional Tables

Table S1. Full length cDNA sequences identified in the National Center for Biotechnology Information (NCBI) database coding for putative diterpene synthases (DTPS) in the *Pinus* species. ORF, open reading frame; bp, base pair

Species	Function	Abbreviation	Accession mRNA sequence	ORF (bp)	Accession protein sequence	Predicted number of amino acids
Pinus banksiana	Levopimaradiene/Abietadiene synthase	Pb DTPS LAS1	JQ240312	2574	AFU73864	857
	Monofunctional diterpene synthase	Pb MDTPS1	JQ240317	2559	AFU73869	852
	Monofunctional isopimaradiene synthase	Pb DTPS mISO1	JQ240313	2631	AFU73865	876
	Monofunctional pimaradiene synthase	Pb DTPS mPIM1	JQ240315	2607	AFU73867	868
Pinus contorta	Levopimaradiene/Abietadiene synthase	Pc DTPS LAS1	JQ240310	2574	AFU73862	857
	Levopimaradiene/Abietadiene synthase	Pc DTPS LAS2	JQ240311	2553	AFU73863	850
	Monofunctional diterpene synthase	Pc MDTPS1	JQ240318	2559	AFU73870	852
	Monofunctional diterpene synthase	Pc MDTPS2	JQ240319	2559	AFU73871	852
	Monofunctional diterpene synthase	Pc MDTPS3	JQ240320	2559	AFU73872	852
	Monofunctional isopimaradiene synthase	Pc DTPS mISO1	JQ240314	2631	AFU73866	876
	Monofunctional pimaradiene synthase	Pc DTPS mPIM1	JQ240316	2607	AFU73868	868
Pinus densiflora	Abietadiene synthase	Pd DTPS ABS1	EU439295	2577	ACC54559	858
Pinus taeda	Diterpene synthase	Pt DTPS LAS1	AY779541	2553	AAX07435	850

Table S2. Forward and Reverse primers used for the isolation of cDNAs and genomic diterpene synthase sequences in *Pinus nigra* subsp. *laricio.* RACE, Rapid Amplification of cDNA Ends

	Phylogentic group	Forward primers 5’→3’	Reverse primers 5’→3’
**Partial cDNA sequences**	Group 1	F1c: CAAGGTTGCACCATCAGACG	R1c: GCCTCTTCCAGCCAGTTGC
	Group 2	F1c: ACGAAACGAGAATTCCCTGA	R1c: AACCGGTTCGAAGAGGAC
	Group 3	F1c: TTGGAGAAGGCGCAACCT	R1c: AGGGACTGGTTCGAATAGG
	Group 4	F1c: GTTCAAGCAACATCGTTGCC	R1c: ACGCAACTGGTTCGAACA
		**RACE 5'**	**RACE 3'**
**Full length cDNA sequences by RACE**	Group 1	R1: CTTCHCCCCAAGAGCCATC<br>R2: CGTCTGATGGTGCAACCTTG<br>R3: TGCCTGGTTATCTTTACCC	F1Race3': ATAGAAGTATGCCAAGGCTGG
	Group 2	R1: CTTCHCCCCAAGAGCCATC<br>R2: AACAGCCGGAATCCTGG<br>R3: TGACTCCACACGCTTCTC	F1Race3': AAGGTGGTGGAAATCATCG
	Group 3	R1: CTTCHCCCCAAGAGCCATC<br>R2: GCTGCTGCCACCTTATTAGA<br>R3: CCTTCTCCAACACCAGCA	F1Race3': AAGGTGGTGGAAATCATCG
	Group 4	R1: CTTCHCCCCAAGAGCCATC<br>R2: CAGGCTTGTGAGATGACATT<br>R3: TTGCTTGAACCTGATCGT	F1Race3': GACCGCAAATACTTGGAACTG
		**Forward primers 5’→3’**	**Reverse primers 5’→3’**
**Genomic sequences**	Group 1	F1g: ATGCCTTCCTCTTCATTG	R1g: GCCTCTTCCAGCCAGTTGC
	Group 2	F1g: ATGGCCATGCCCTCCTCT	R1g: AACCGGTTCGAAGAGGAC
	Group 3	F1g: ATGGCCATGCCTTCGTAC	R1g: AGGGACTGGTTCGAATAGG
	Group 4	F1g: ATGGCCATGCCTTTGTG	R1g: ACGCAACTGGTTCGAACA

Table S3. Amino acid sequence identity matrix comparing the diterpene synthase (DTPS) candidate genes from *Pinus nigra* subsp. *laricio* (in red) with previously characterized DTPSs from other *Pinus* species, namely *P. taeda* (Pt), *P. contorta* (Pc) and *P. banksiana* (Pb).

	1	2	3	4	5	6	7	8	9	10	11	12	13	14	15	16
1) Pnl_DTPS1	100															
2) Pb_DTPS_LAS1	98.5	100														
3) Pc_DTPS_LAS1	98.2	99.4	100													
4) Pc_DTPS_LAS2	98.0	99.4	99.6	100												
5) Pt_DTPS_LAS1	98.4	99.6	99.4	99.3	100											
6) Pnl_DTPS2	64.3	64.5	64.2	64.6	65.0	100										
7) Pc_MDTPS_1	64.2	64.3	64.1	64.5	64.8	95.2	100									
8) Pc_MDTPS_2	64.6	64.7	64.5	64.9	65.1	95.4	96.8	100								
9) Pc_MDTPS_3	64.7	64.8	64.6	65.0	65.2	95.3	96.8	97.5	100							
10) Pb_MDTPS_1	64.1	64.2	64.0	64.4	64.6	95.4	97.8	97.7	97.7	100						
11) Pnl_DTPS3	66.6	66.6	66.5	66.9	67.0	75.0	74.9	74.8	74.7	74.3	100					
12) Pb_DTPS_mISO1	67.4	67.4	67.3	67.7	67.8	75.7	75.9	75.9	75.5	75.4	97.0	100				
13) Pc_DTPS_mISO1	67.1	67.1	66.9	67.4	67.5	75.3	75.3	75.3	74.9	74.8	96.5	99.2	100			
14) Pnl_DTPS4	63.0	63.2	63.1	63.4	63.5	74.2	74.0	74.3	73.6	73.7	79.2	79.7	79.2	100		
15) Pb_DTPS_mPIM1	63.5	63.8	63.7	64.0	64.1	73.4	73.4	73.7	73.1	73.0	79.8	80.0	79.5	93.8	100	
16) Pc_DTPS_mPIM1	63.5	63.7	63.6	63.9	64.0	73.8	73.8	74.2	73.5	73.5	80.0	80.3	79.7	93.8	98.6	100

Additional Figures

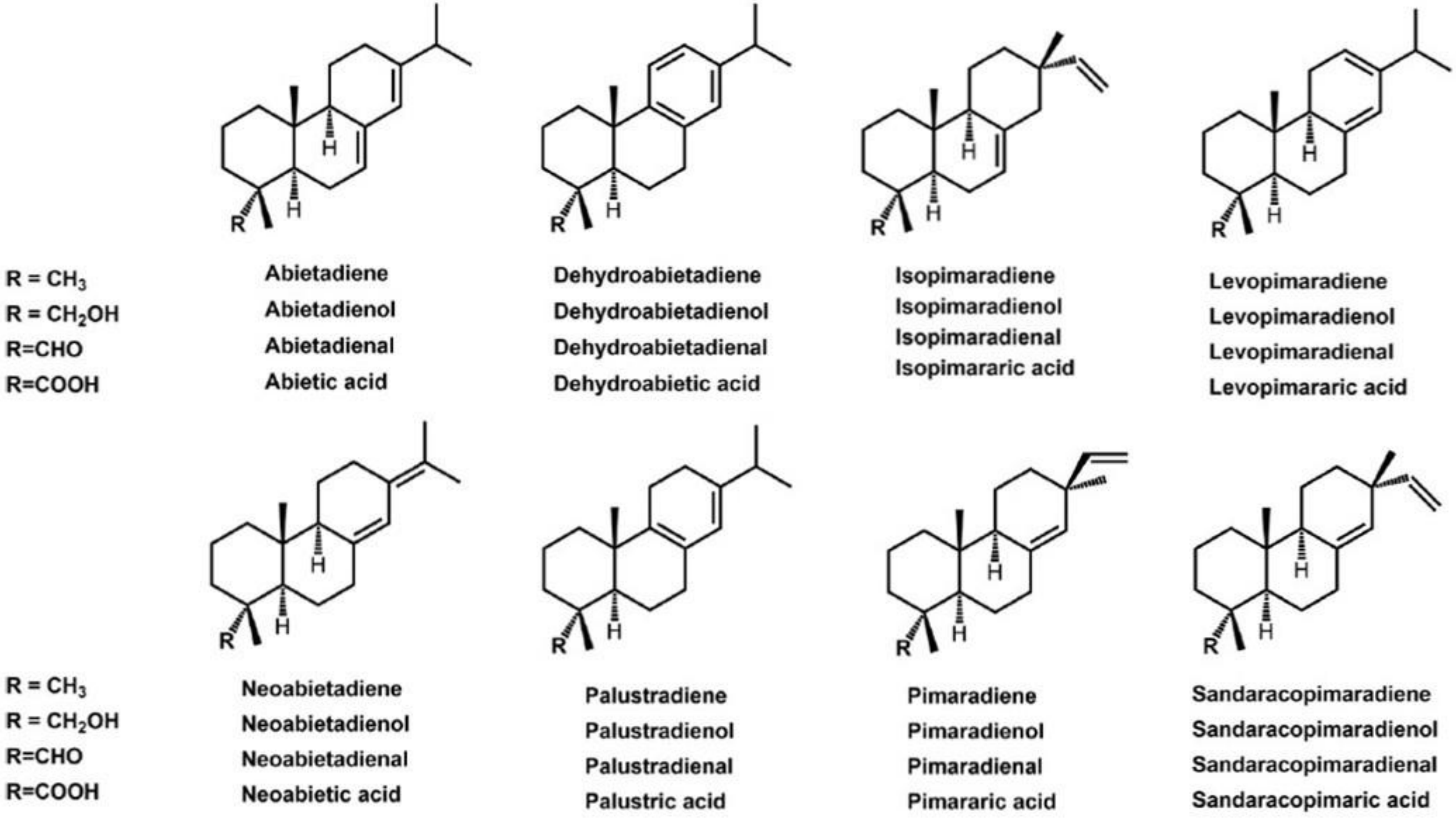

Figure S1. Chemical structures of the most represented diterpenoids in *Pinus* spp. [R = CH_3 olefins constituents; R = CH_2OH alcoholic constituents; R = CHO aldehydic constituents; R = COOH diterpene resin acid (DRA) constituents]. (Adapted from Turner et al., 2019).

[Turner, G. W.; Parrish, A. N.; Zager, J. J.; Fischedick, J. T.; Lange, B. M. Assessment of Flux through Oleoresin Biosynthesis in Epithelial Cells of Loblolly Pine Resin Ducts. *Journal of Experimental Botany* **2019**, *70* (1), 217–338. https://doi.org/10.1093/jxb/ery338.

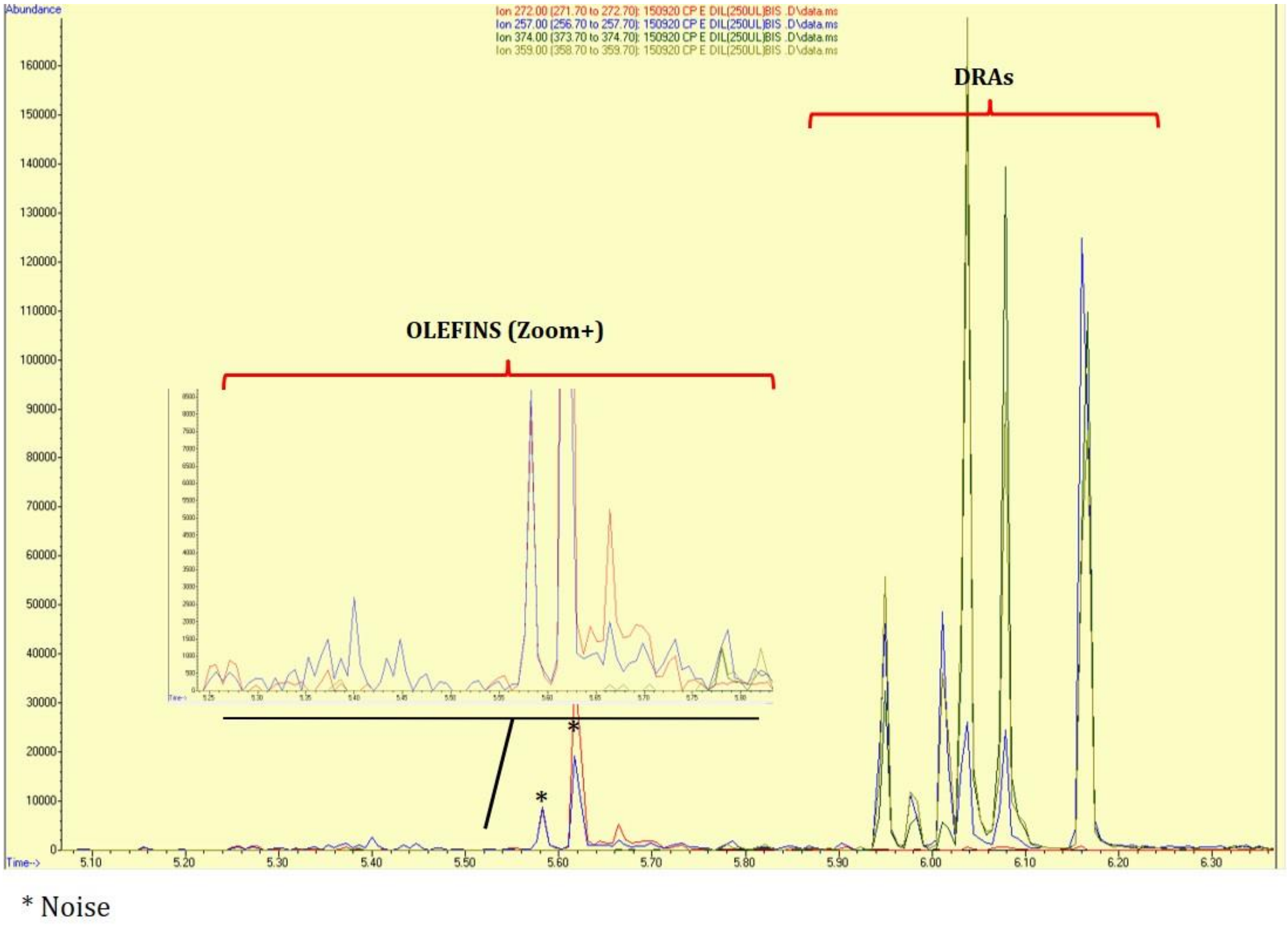

Figure S2. A representative example of the quantitative relationships among acidic (diterpene resin acids, DRAs) and neutral (olefins) components of the diterpenes extracted from *Pinus nigra* subsp. *laricio* (Calabrian pine) tissues, visualized by overlapping GC-MS ion chromatograms at selected m/z, i.e., 374/359 for DRA and 272/257 for olefins (magnified inset on the bottom left side of the item).

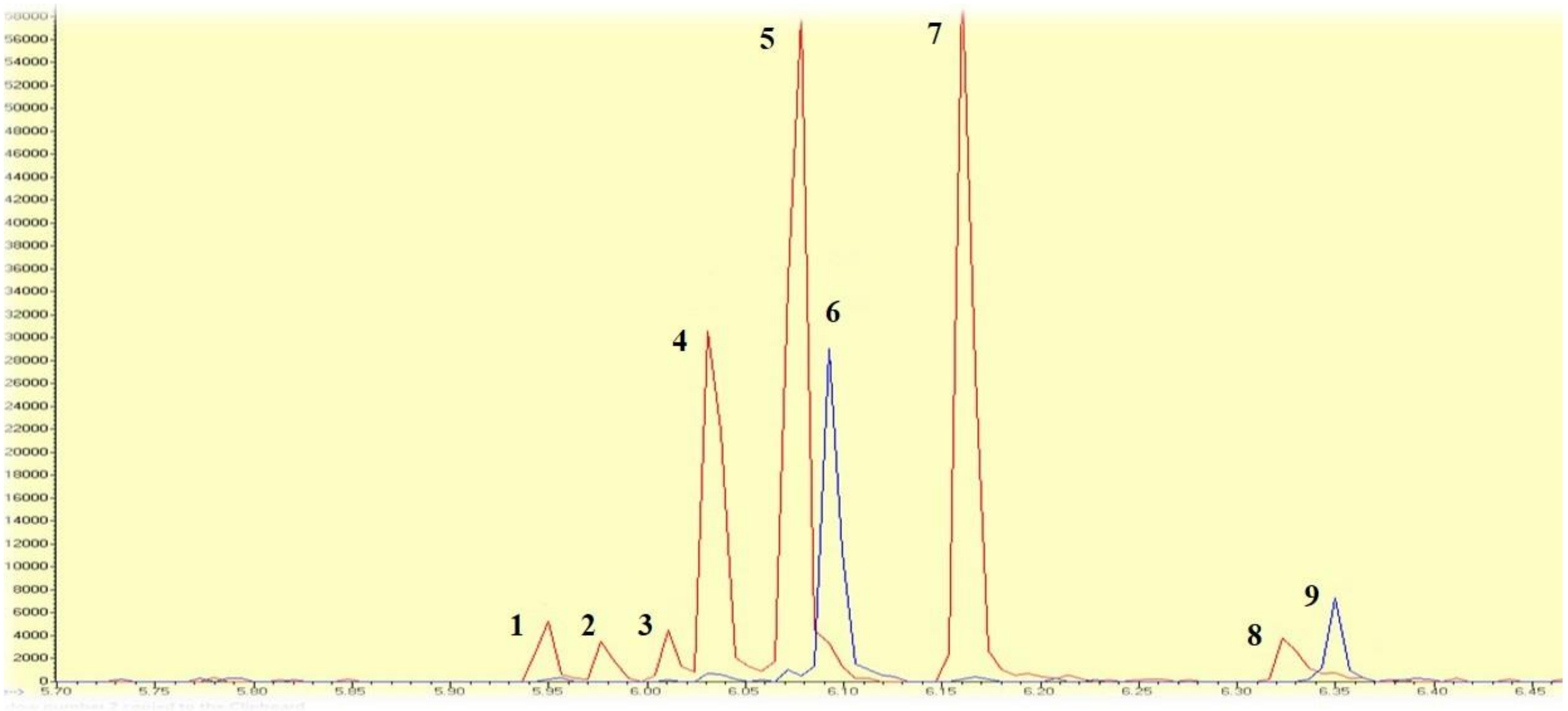

Figure S3. A representative GC-MS profile of the diterpene resin acids extracted from the leader stem of Calabrian pine. The single ion monitoring at m/z 374 (red line, non-dehydrogenated species) was overlapped with the single ion monitoring at m/z 372 (blue line, dehydrogenated species). (1), pimaric acid; (2), sandaracopimaric acid; (3), isopimaric acid; (4), palustric acid; (5), levopimaric acid; (6), dehydroabietic acid; (7), abietic acid; (8), neoabietic acid; (9), non-identified dehydroisomer.

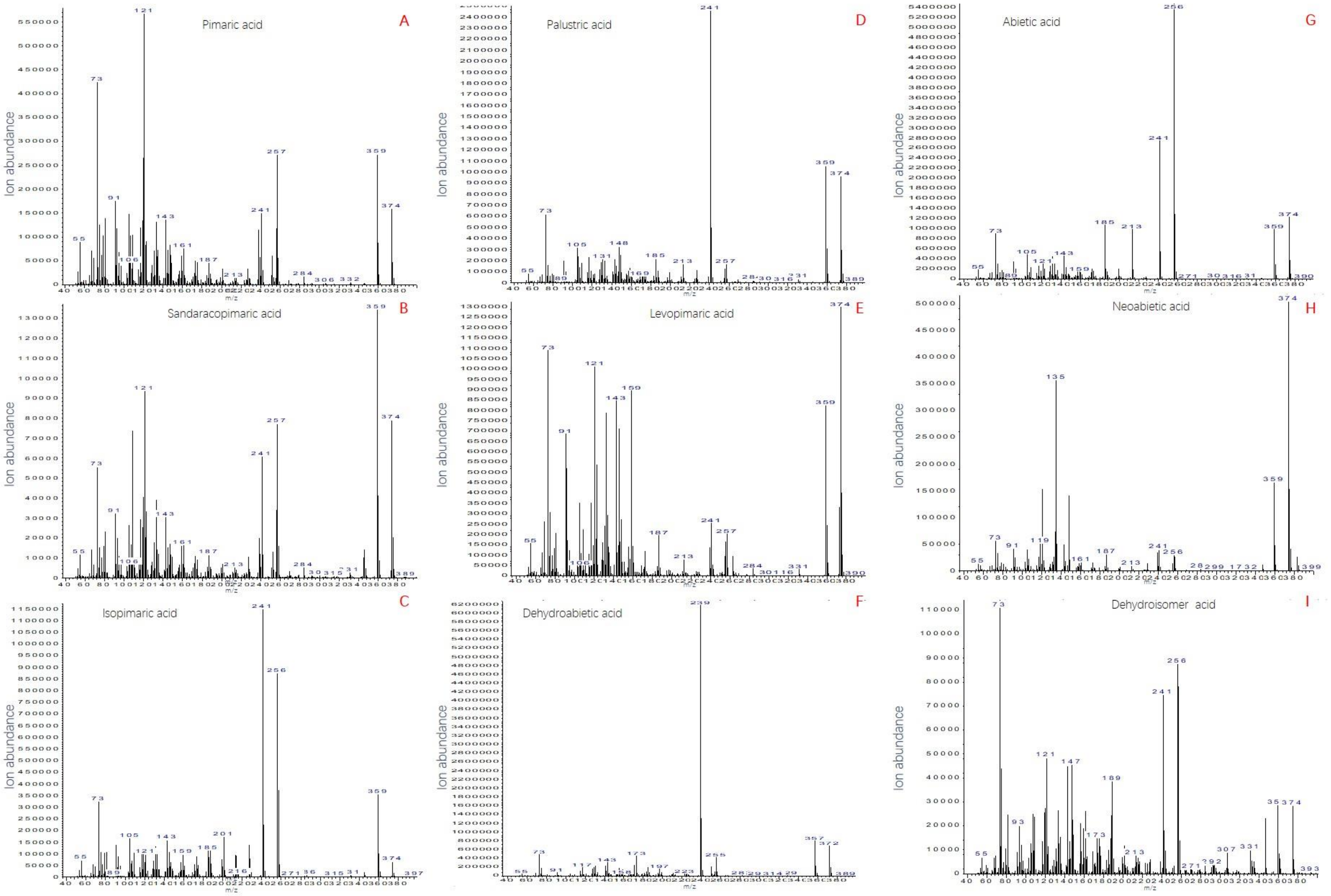

Figure S4. Mass spectra of the nine diterpene resin acids identified in *Pinus nigra* subsp. *laricio*. Pimaric acid (A), Sandaracopimaric acid (B), Isopimaric acid (C), Palustric acid (D), Levopimaric acid (E), Dehydroabietic acid (F), Abietic acid (G), Neoabietic acid (H) and Dehydroisomer acid (I).

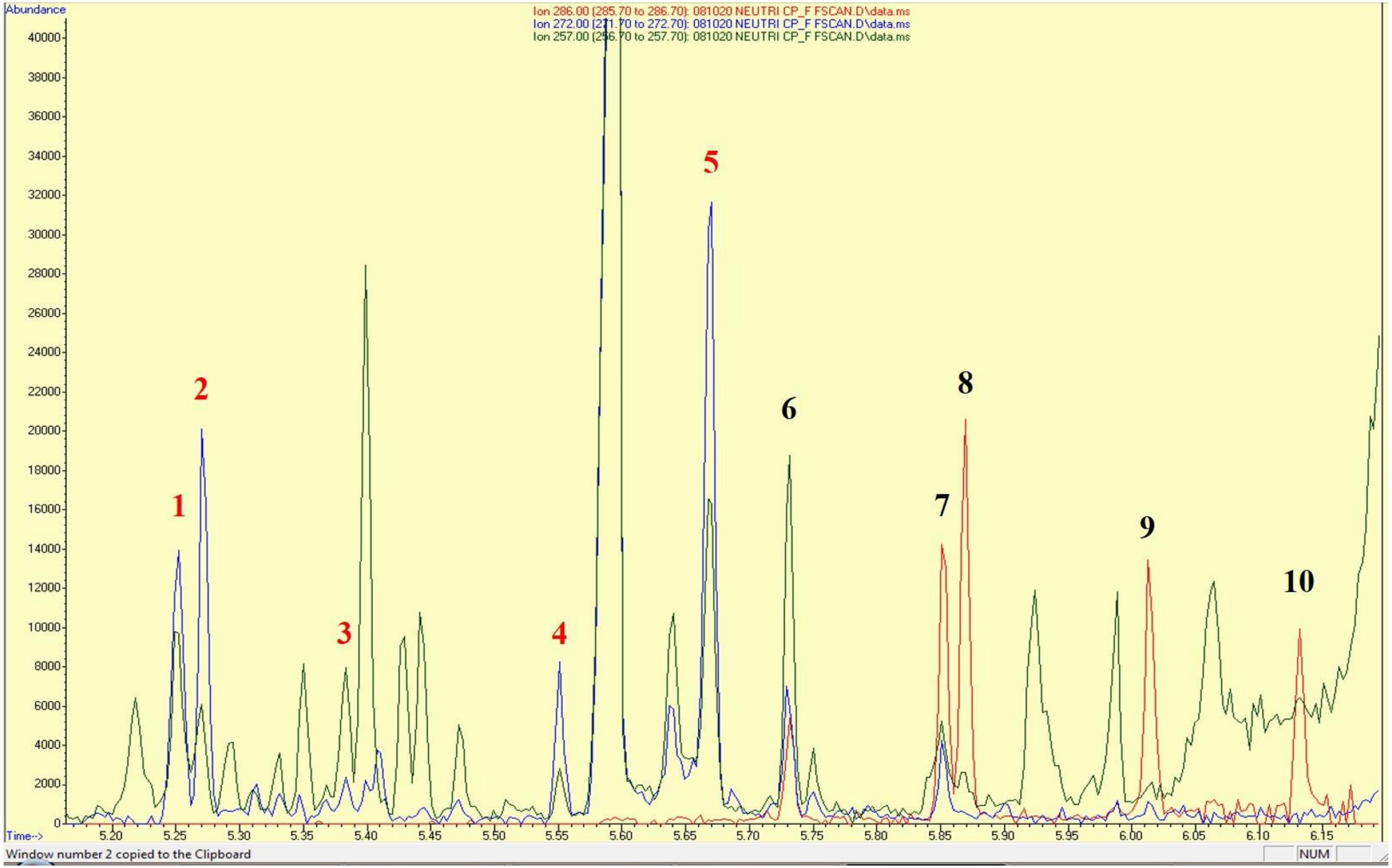

Figure S5. A representative GC-MS profile of the diterpene olefins and aldehydes in the extract obtained from leader stem tissue of Calabrian pine. The selected m/z 272 and 257 for diterpene olefins (red number above peaks) were overlapped with the selected m/z 286 for aldehydes (black number above peaks). (1) sandaracopimaradiene; (2) levopimaradiene; (3) palustradiene; (4) abietadiene; (5) neoabietadiene; (6) sandaracopimaradienal; (7) palustradienal; (8) isopimaradienal; (9) abieta-dienal; (10) neoabietadienal.

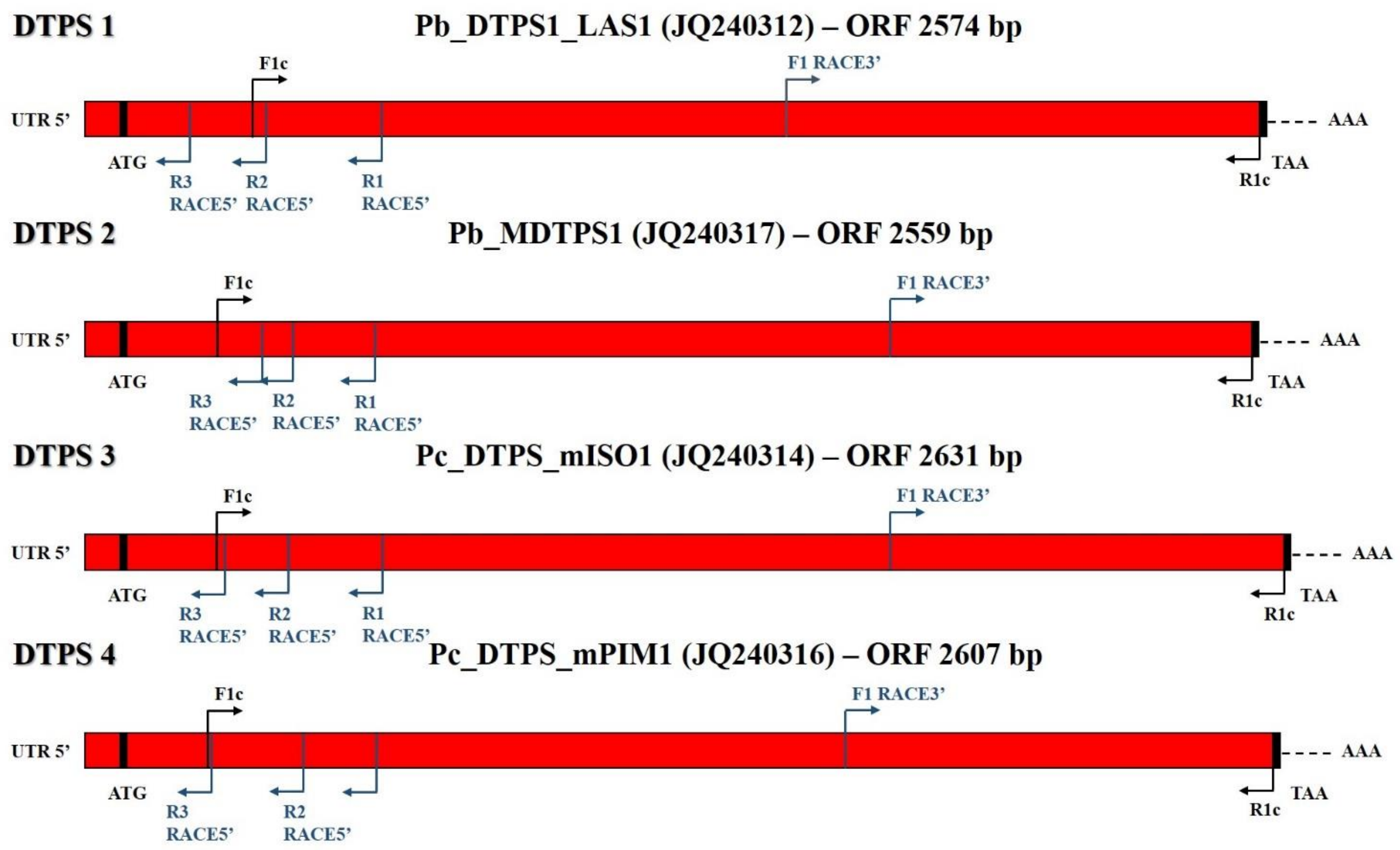

Figure S6. Schematic representation of the full length cDNAs of four representative diterpene synthase (*DTPS*) members of the phylogenetic d3 clade of genes, two from *Pinus banksiana* (Pb) and two from *Pinus contorta* (Pc) and the positions of their Forward and Reverse primers used in the present study for the isolation of the partial transcripts coding for the orthologous genes (F1c/R1c) in Calabrian pine. The position of the specific primers used for 5' and 3' RACE (Rapid Amplification of cDNA Ends) extensions of partial transcripts of Calabrian pine *DTPS* (groups 1-4) are indicated in blue. UTR, untranslated region

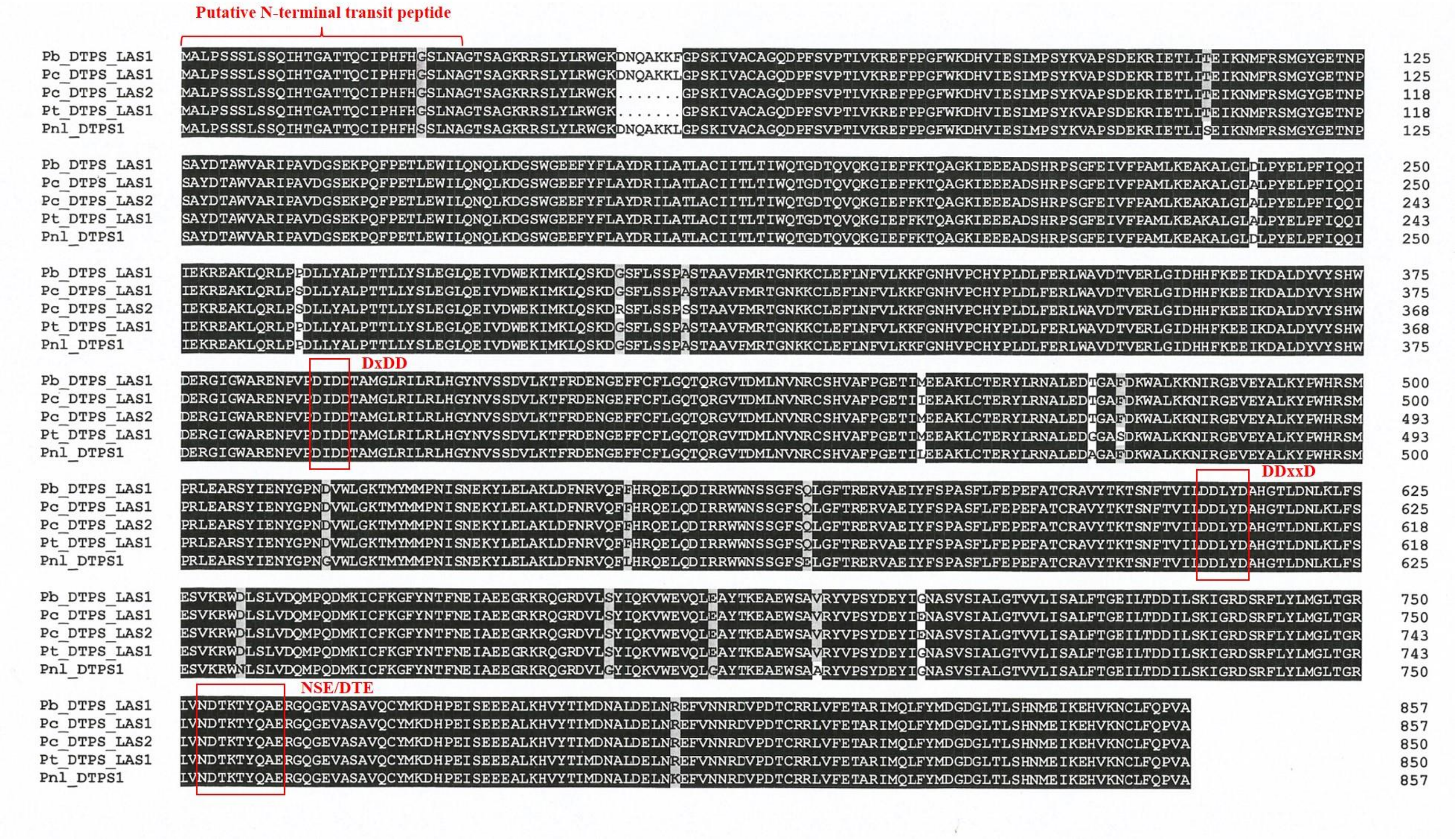

Figure S7. Alignment of deduced amino acid sequences of diterpene synthase (DTPS) belonging to the phylogenetic group 1. Amino acid residues with black background indicate highly conserved regions, while amino acid residues which are identical in more than 50% of the proteins are in grey background. The DTPS class II (DxDD) and class I (DDxxD, NSE/DTE) signature motifs are indicated. Pb, *Pinus banksiana*; Pc, *Pinus contorta*; Pt, *Pinus taeda*; Pnl, *Pinus nigra* subsp. *laricio*.

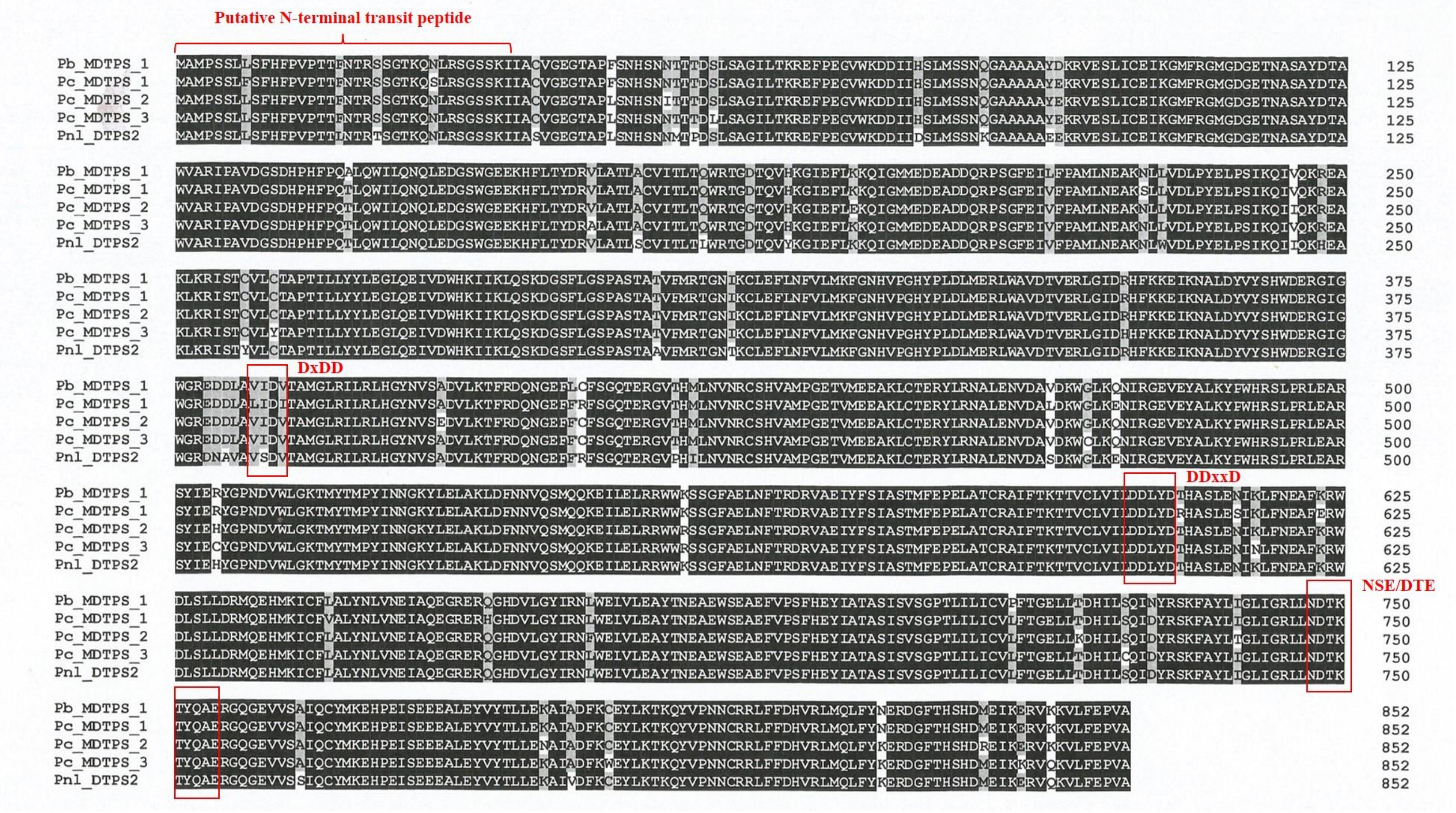

Figure S8. Alignment of deduced amino acid sequences of diterpene synthase (DTPS) belonging to the phylogenetic group 2. Amino acid residues with black background indicate highly conserved regions, while amino acid residues which are identical in more than 50% of the proteins are in grey background. The DTPS class II (DxDD) and class I (DDxxD, NSE/DTE) signature motifs are indicated. Pb, Pc, and Pnl as in Fig. S7

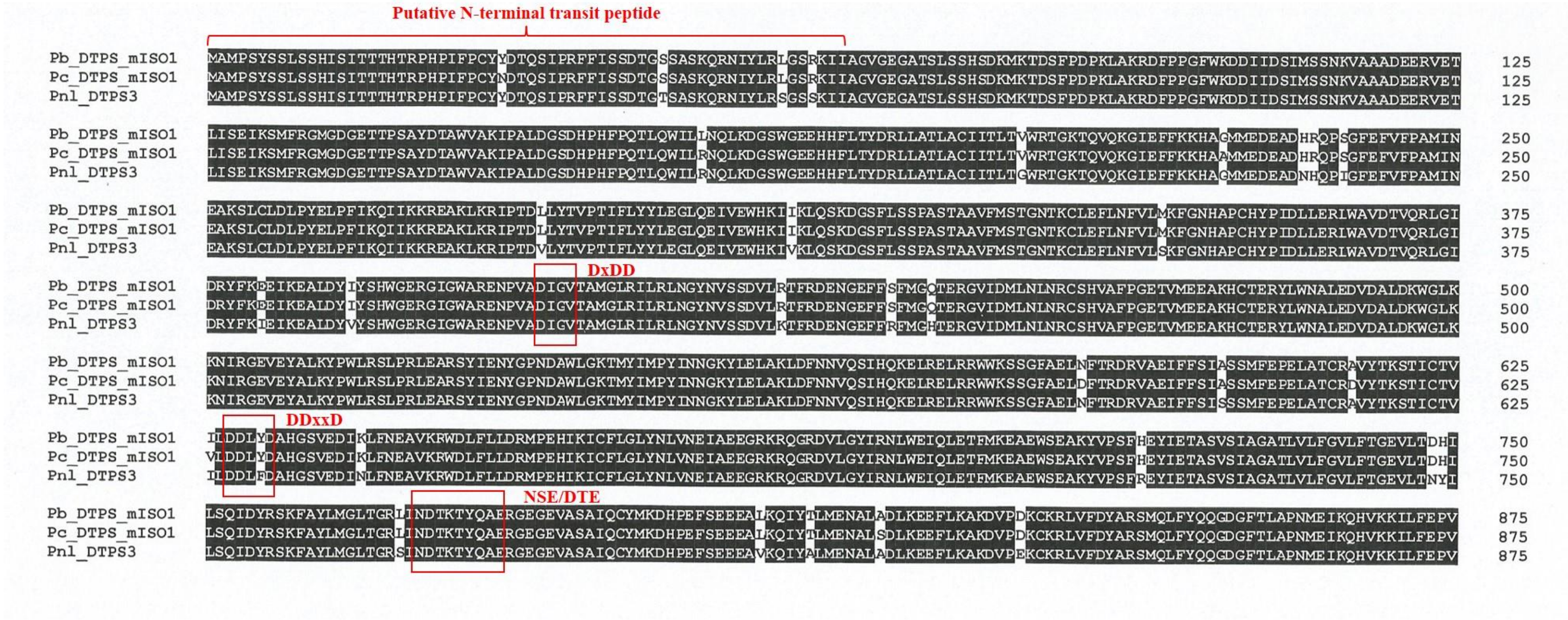

Figure S9. Alignment of deduced amino acid sequences of diterpene synthase (DTPS) belonging to the phylogenetic group 3. Amino acid residues with black background indicate highly conserved regions, while amino acid residues which are identical in more than 50% of the proteins are in grey background. The DTPS class II (DxDD) and class I (DDxxD, NSE/DTE) signature motifs are indicated. Pb, Pc, and Pnl as in Fig. S7

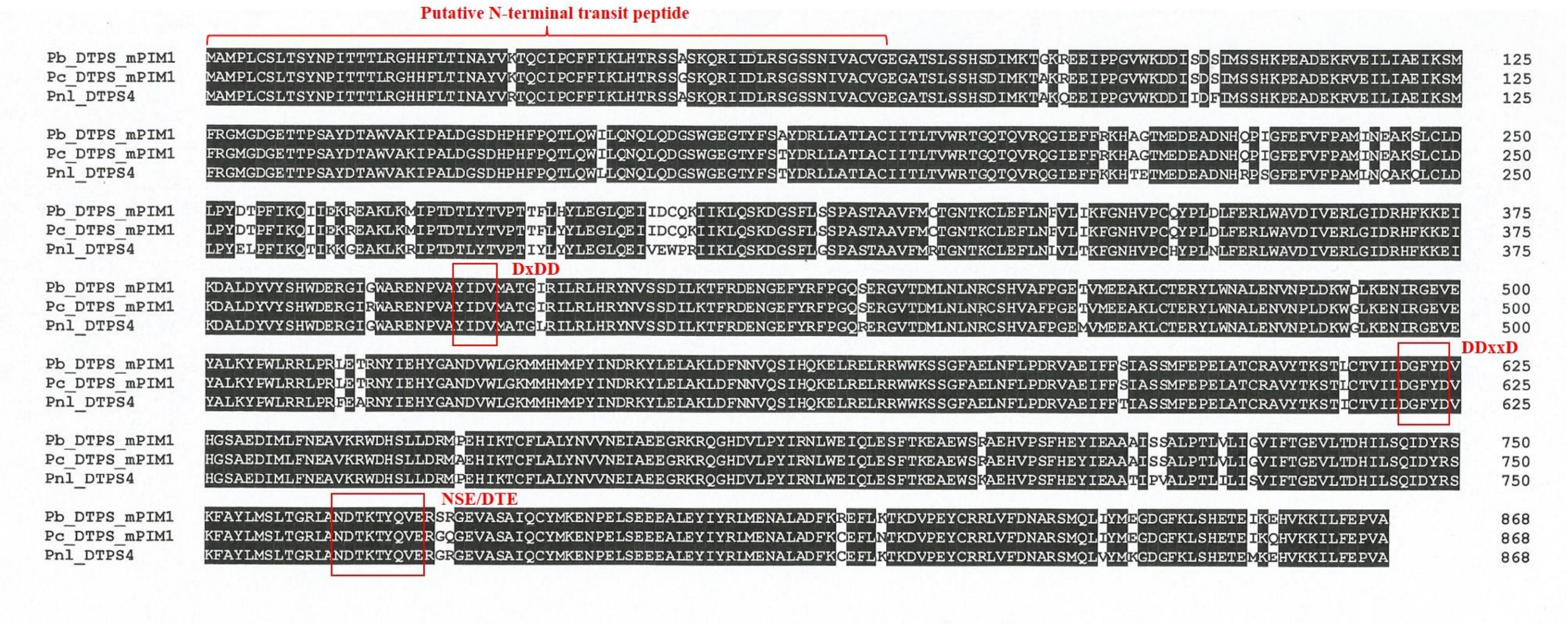

Figure S10. Alignment of deduced amino acid sequences of diterpene synthase (DTPS) belonging to the phylogenetic group 4. Amino acid residues with black background indicate highly conserved regions, while amino acid residues which are identical in more than 50% of the proteins are in grey background. The DTPS class II (DxDD) and class I (DDxxD, NSE/DTE) signature motifs are indicated. Pb, Pc, and Pnl as in Fig. S7

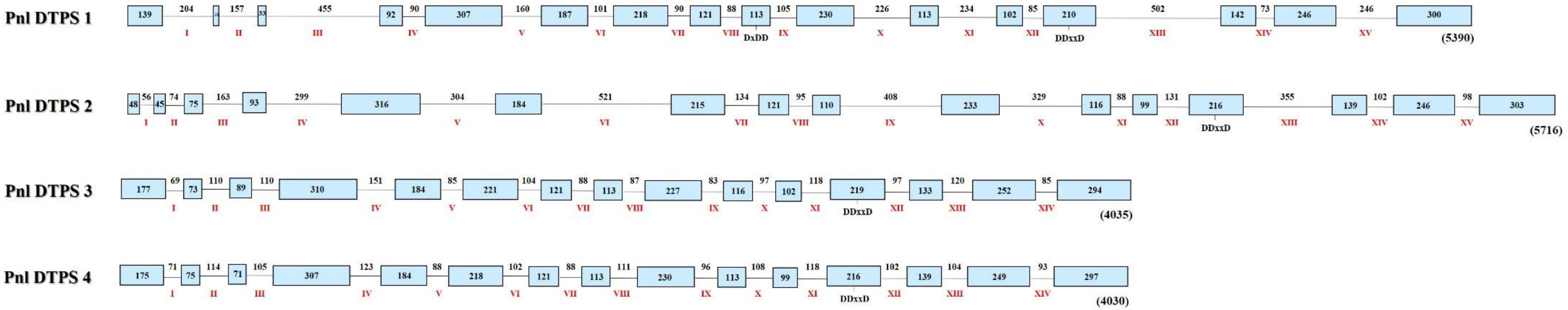

Figure S11. Schematic representation of the exon/intron structures of the four diterpene synthase (*DTPS*) genes isolated from Calabrian pine (Pnl) in the present study. For both exons (blue boxes) and introns (black lines) the lengths in bp are indicated. Introns were numbered (Roman numerals) starting from the 5' end of each genomic sequences.
